# Individual patient oesophageal cancer 3D models for tailored treatment

**DOI:** 10.18632/oncotarget.12500

**Published:** 2016-10-06

**Authors:** John H. Saunders, David Onion, Pamela Collier, Matthew S. Dorrington, Richard H. Argent, Philip A. Clarke, Alex M. Reece-Smith, Simon L. Parsons, Anna M. Grabowska

**Affiliations:** ^1^ Cancer Biology Unit, Division of Cancer & Stem Cells, School of Medicine, University of Nottingham, Nottingham, UK; ^2^ Department of Upper GI Surgery, City Hospital Campus, Nottingham University Hospitals NHS Trust, Nottingham, UK

**Keywords:** oesophageal cancer, personalised treatment, 3D models, chemotherapy, tumour microenvironment

## Abstract

**Background:**

A model to predict chemotherapy response would provide a marked clinical benefit, enabling tailored treatment of oesophageal cancer, where less than half of patients respond to the routinely administered chemotherapy.

**Methods:**

Cancer cells were established from tumour biopsies taken from individual patients about to undergo neoadjuvant chemotherapy. A 3D-tumour growth assay (3D-TGA) was developed, in which cancer cells were grown with or without supporting mesenchymal cells, then subjected to chemo-sensitivity testing using the standard chemotherapy administered in clinic, and a novel emerging HDAC inhibitor, Panobinostat.

**RESULTS:**

Individual patients cancer cells could be expanded and screened within a clinically applicable timescale of 3 weeks. Incorporating mesenchymal support within the 3D-TGA significantly enhanced both the growth and drug resistance profiles of the patients cancer cells. The *ex vivo* drug response in the presence, but not absence, of mesenchymal cells accurately reflected clinical chemo-sensitivity, as measured by tumour regression grade. Combination with Panobinostat enhanced response and proved efficacious in otherwise chemo-resistant tumours.

**Conclusions:**

This novel method of establishing individual patient oesophageal cancers in the laboratory, from small endoscopic biopsies, enables clinically-relevant chemo-sensitivity testing, and reduces use of animals by providing more refined *in vitro* models for pre-screening of drugs. The 3D-TGA accurately predicted chemo-sensitivity in patients, and could be developed to guide tailored patient treatment. The incorporation of mesenchymal cells as the stromal cell component of the tumour micro-environment had a significant effect upon enhancing chemotherapy drug resistance in oesophageal cancer, and could prove a useful target for future drug development.

## INTRODUCTION

There are over 8,000 new diagnoses of oesophageal cancer per year in the UK and near 500,000 worldwide. [1] Mortality remains high, with a 5-year overall survival of only 13%. [1] In the UK and much of Europe, routine treatment for potentially curable patients is neo-adjuvant chemotherapy and resectional surgery followed by adjuvant chemotherapy. [2] However, the response rate to neo-adjuvant chemotherapy is only 40%, so over half of patients do not benefit whilst suffering toxic chemotherapy-related side-effects. [3] A model to understand the mechanisms of chemo-resistance in tumours and that can potentially predict which patients are most likely to benefit from chemotherapy would provide a marked clinical advantage and an opportunity for personalised treatment of oesophageal cancer. [4]

We have previously described a pre-clinical tumour model (3D tumour growth assay, 3D-TGA) that allows chemotherapeutic drug testing in a more accurate and clinically-relevant setting, using ‘close-to-patient’ cells isolated from patient-derived xenografts. [5] Incorporating extracellular matrix and mesenchymal support restores both direct and paracrine tumour-stroma interactions which are known to influence drug resistance. [6, 7]

Here, we have modified the 3D-TGA to allow it to be used for cells derived from small biopsies such as those taken endoscopically prior to chemotherapy treatment of oesophageal cancer. This would allow the evaluation of the chemo-naïve tumour within a clinically-relevant timescale of 3 weeks, whilst the patient is progressing through the histological diagnosis, multicomponent staging and cancer MDT pathway [8], before referral for consideration of neo-adjuvant chemotherapy. The assay is amenable to pharmacological testing in a 384-well format allowing numerous drugs and combinations to be tested simultaneously. The clinical validity was assessed by comparing the chemo-sensitivity measured in the 3D-TGA with the actual clinical response, as measured by the Mandard tumour regression grade (TRG). [9]

## RESULTS

### Close-to-patient oesophageal cancer cells can be established *in vitro* using a feeder layer culture system and grown in the 3D-TGA

79 chemotherapy-naïve tumour biopsy samples were obtained from the 70 patients recruited. A cohort of 30 patients and their tissue was used in the novel method development phase of the study and did not generate patient cancer cells. Using the feeder layer method, individual patient *in vitro* cancer cell cultures were established reliably in a subsequent group of 28/40 patients (70%); with the other 12 patient's tumour cultures excluded for technical reasons (see online Supplementary Figure S1). There was no apparent difference in oncological or demographic characteristics between those that did / did not establish (see online Supplementary Table S3).

Clinical inclusion criteria (oesophageal adenocarcinoma; completion of 3 full-dose cycles of ECF neoadjuvant chemotherapy; definitive surgery and TRG assigned) were necessary to ensure accurate correlation between the clinical chemo-sensitivity in patients as measured by TRG, and *in vitro* chemo-sensitivity as assessed by the 3D-TGA. Patient reasons (e.g. advanced disease requiring palliation) and oncological causes (e.g. non-completion of chemotherapy) requiring study exclusion, resulted in a final group of 12 samples from nine patients who underwent the detailed chemotherapeutic analysis in this study (see online Supplementary Figure S1). Five of these nine patients had a matched, chemotherapy-exposed resected tumour established *in vitro* which also underwent chemo-sensitivity analysis. The baseline demographic, surgical and oncological details for these nine patients with samples established from chemotherapy-naïve biopsies who met these inclusion criteria were recorded (Table 1), have a similar distribution of grade and aggressiveness, and are comparable to a standard clinical cohort presenting with disease amenable to neoadjuvant chemotherapy and surgery with curative intent. [10]

**Table 1:** Patient demographics, tumour staging and treatment

Patient ID	Demographics & Diagnosis				Chemotherapy & Surgery					Histopathology & Tumour Regression				
	Gender	Age at diagnosis	Tumour site	cTNM (all M0)	Cycles of NeoA ECF	NeoA dose reduction	NeoA cycle delay	NeoA complication	Procedure	pTNM (all M0)	Positive / Resected Nodes	Involved margins	Stage	TRG
Oes1	M	77	Lower third	T3 N0	3	no	nil	none	ILO	T0 N0	0/12	no	0	1
Oes2	M	55	Lower third	T3 N2	3	no	nil	none	ILO	T3 N1	2/30	no	IIIA	3
Oes3	M	45	GOJ	T3 N0	3	no	nil	none	ILO	T4a N1	1/23	no	IIIC	3
Oes4	F	53	GOJ	T3 N2	3	no	nil	none	ILO	T4a N0	0/7	CRM+	IIIA	4
Oes5	M	58	GOJ	T3 N2	3	no	nil	none	ILO	T3 N3	8/18	CRM+	IIIC	4
Oes6	M	72	GOJ	T3 N2	3	no	1 week	Diarrhoea, N+V, fatigue	ILO	T3 N0	0/16	CRM+	IIB	4
Oes7	M	75	Lower third	T3 N1	3	no	2 week	AKI, mucositis	ILO	T3 N0	0/22	no	IIB	5
Oes8	M	62	Lower third	T3 N1	3	no	nil	Neutropenia, N+V (during 3rd cycle)	ILO	T3 N2	4/17	CRM+	IIIB	5
Oes9	M	67	GOJ	T3 N0	3	no	nil	PPE	ILO	T3 N3	12/18	CRM+	IIIC	4

Footnote: Abbreviations: GOJ: gastro-oesophageal-junction, cTNM: clinical Tumour, Node & Metastasis at initial staging, NeoA: Neoadjuvant chemotherapy, ECF: Epirubicin, Cisplatin, 5-FU, N+V: nausea & vomiting, AKI: acute kidney injury, PPE: Palmar-Plantar Erythrodysesthesia, ILO: Ivor-Lewis Oesophagectomy, pTNM; pathological TNM histologically at resection, CRM+: circumferential resection margin positive, TRG: Mandard tumour regression grade.

**Figure 1:**
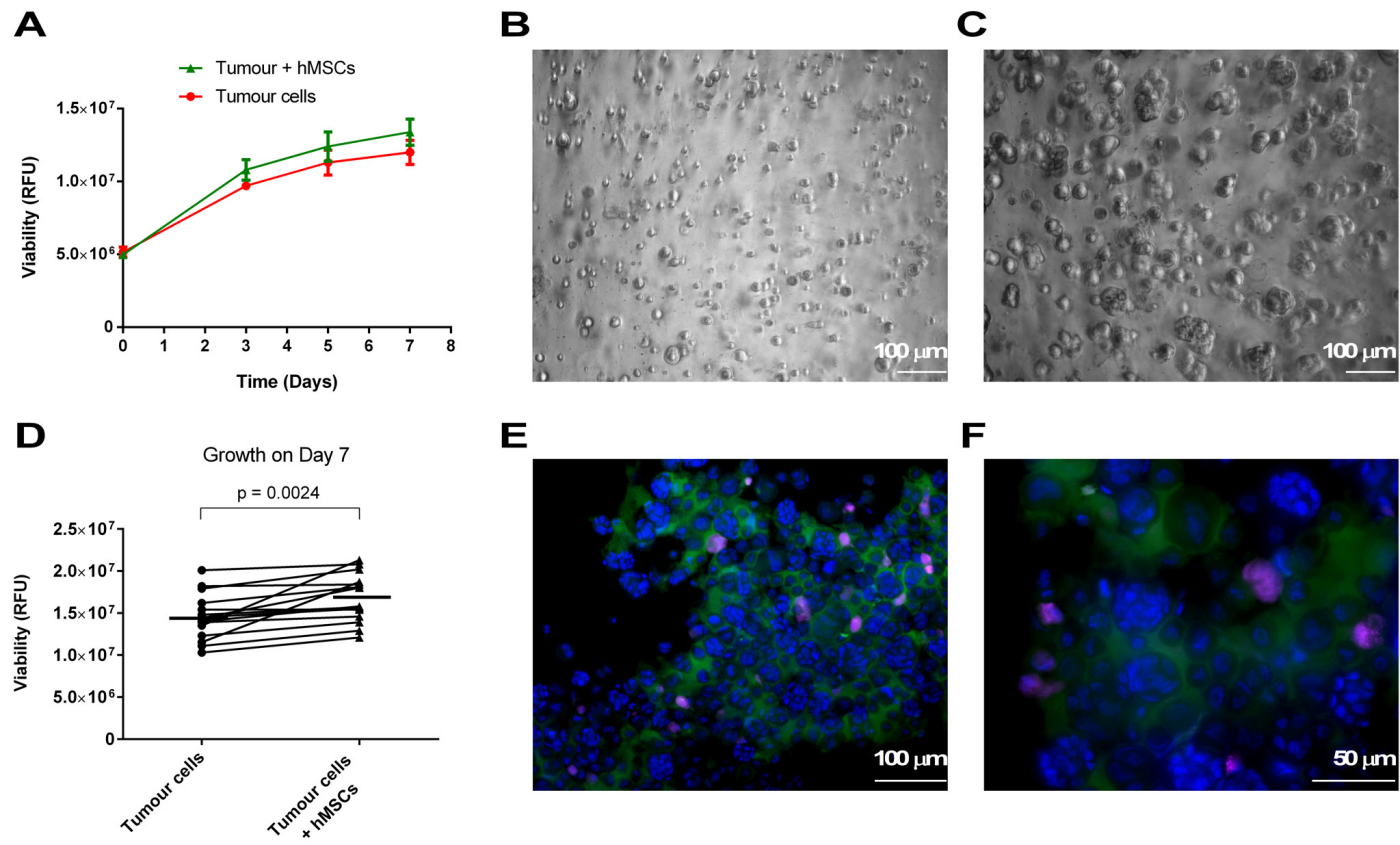
Growth in the 3D-TGA Tumour cells were seeded in 3D-TGA with and without hMSCs. Growth of close-to-patient cells was determined in 3D-TGA over the 7 day assay using the alamarBlue assay. **A**. Progressive growth of tumour cells was monitored in 6 replicate wells immediately after establishment in 3D (day 0) and on days 3, 5 and 7. **B**., **C**. 3D cultures of live cells were imaged by wide-field microscopy with DIC at day 0 **B**. and day 7 **C**.. Images are extended depth of field projection of multiple z-stacks taken of Oes5R. **D**. The day 7 mean peak values from the growth curves were plotted in pairs, demonstrating a small but significant increase in growth in the models that incorporate mesenchymal support. **E**., **F**. Cell clusters with mCherry-labelled hMSCs (purple) at day 7 were extracted to glass slides and stained with anti-TFF3-AlexaFluor488 (green) and counter-stained with DAPI (blue), and imaged by fluorescence microscopy at x20 **E**. and x40 **F**. magnification.

Mean age on presentation was 63 years, with a male predominance 89% (*n* = 8) and similar cTNMs of T3 N0-2 within the group, and stages IIB to IIIC. The proportion (33%, *n* = 3) of chemotherapy sensitive tumours (TRG 1-3), was broadly comparable to that seen in clinical practice (40%). [10] There was no significant difference between the mean time (26 and 21 days for the TRG 1-3 and TRG 4-5 cancers respectively) to develop each patient tumour into an established *in vitro* patient cancer cell culture of sufficient volume for laboratory experimentation ( > 1 x10^7^ cells). When grown in the 3D-TGA each individual close-to-patient cell culture grew in reproducible fashion (Figure 1A), with some variation in growth rate between the different patient lines, and developed into multicellular cancer cell clusters (Figure 1B, 1C). The growth of the hMSCs was minimal compared to the cancer cells (see online Supplementary Figure S2), so did not affect overall growth measurement by alamarBlue. In co-culture, the cancer cell clusters displayed a small but significant increase in growth (*p* < 0.05) compared to those without mesenchymal support (Figure 1D).

### Cultured patient cancer cell phenotype mirrors primary tumour tissue

**Figure 2:**
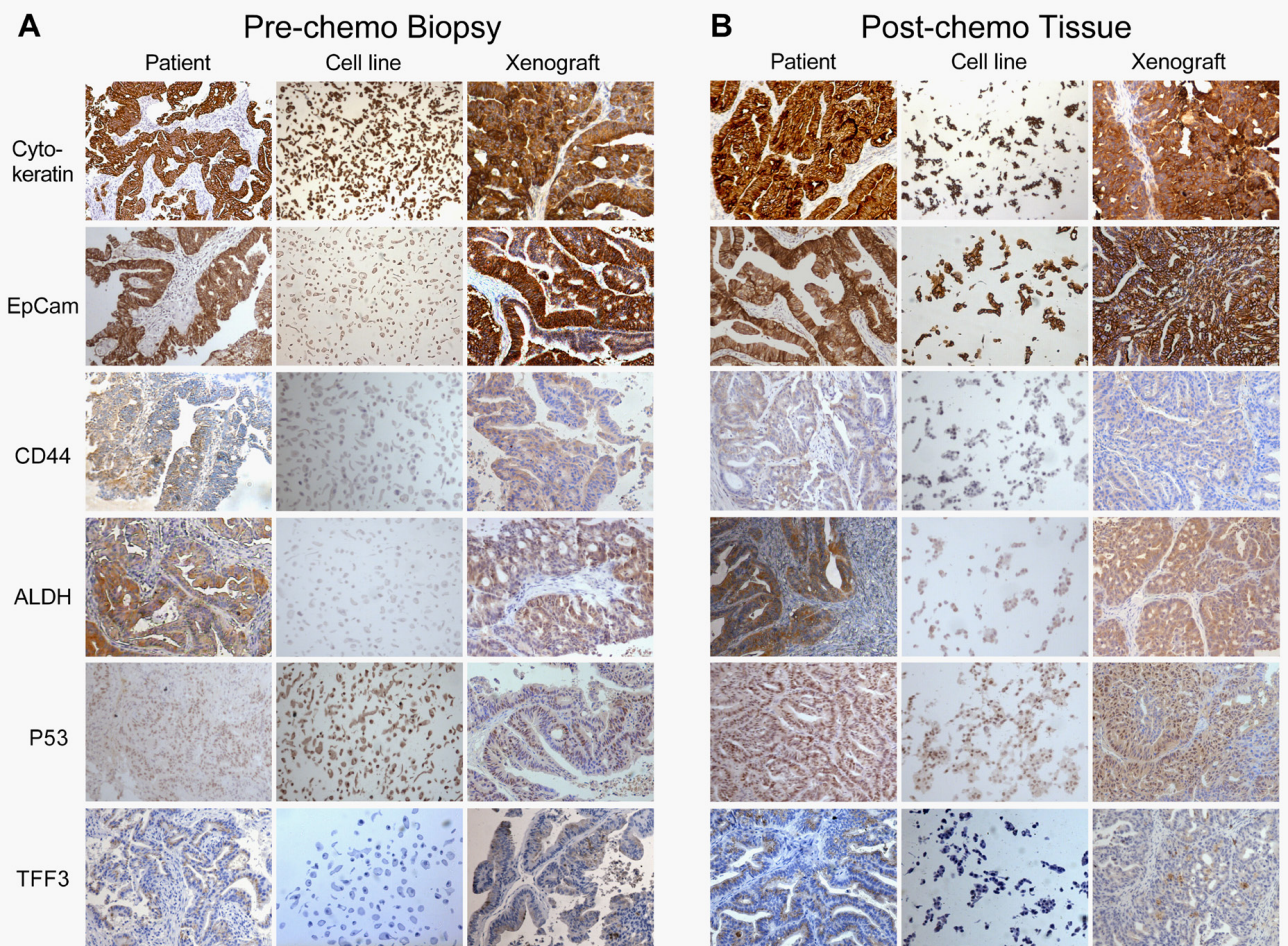
Histology IHC analysis of **A**. pre-chemotherapy biopsy and **B**. matched post-chemotherapy tumour tissue, and their subsequently generated close-to-patient cells and xenograft model, all from patient Oes7. FFPE sections were stained with antibodies against Cytokeratin, EpCam, CD44, ALDH, P53 and TFF3, using standard IHC techniques and visualised with Leica DMLB bright-field microscope at x10 magnification.

The phenotype of the close-to-patient cancer cells was compared with the corresponding primary patient cancer by IHC (Figure 2 and Table 2). Like the primary patient tissues, all were Cytokeratin, EpCam and p53 positive, confirming respectively the epithelial nature of the cells; their derivation from transformed metaplastic columnar-type epithelium (rather than adjacent normal squamous oesophageal epithelium); and neoplastic phenotype, as oesophageal p53 staining is not found in non-dysplastic Barrett's Oesophagus. [11, 12] Trefoil Factor 3 (TFF3) is involved in protection, maintenance and repair of the intestinal mucosa, [13] specific for cells with an intestinal phenotype, [14] and is a reliable marker of metaplastic change in the oesophagus. [15] It was present in all of the primary patient cancer tissue, but absent in the feeder layer culture of the close-to-patient cancer cells, however expression was restored in both the 3D-TGA with mesenchymal support (Figure 1E, 1F) and xenografts (Figure 2). The potential oesophageal cancer stem cell (CSC) markers CD44 [16, 17] and ALDH [18, 19] were present in 11/17 (64.7%) and 13/17 (76.5%) of the samples respectively, and both were present in 9/17 (52.9%) of the samples (Table 2). Expression in the primary patient tumour was reflected in the corresponding close-to-patient cells and xenografts (Figure 2), suggesting maintenance of cells with a CSC-like phenotype in culture. Although numbers are small, the presence of ALDH and CD44 (either individually or together) did not relate to stage of disease, pre- or post-chemotherapy tissue status, the TRG, or *in vitro* growth.

**Table 2:** Characteristics of each patient, and corresponding close-to-patient cells, pre & post chemotherapy

IHC Tissue		Patient						Close-to-patient cells					
Study ID	TRG	Cyto	EpCam	CD44	ALDH	p53	TFF3	Cyto	EpCam	CD44	ALDH	p53	TFF3
**Oes1B**	1	+	+	-	+	+	+	+	+	-	+	+	-
**Oes2B**	3	+	+	-	-	+	+	+	+	-	-	+	-
**Oes3B**	3	+	+	-	+	+	+	+	+	-	+	+	-
**Oes4B**	4	+	+	+	+	+	+	+	+	+	+	+	-
**Oes4R**	4	+	+	+	+	+	+	+	+	+	+	+	-
**Oes5B(i)**	4	+	+	+	+	+	+	+	+	+	+	+	-
**Oes5B(ii)**	4	+	+	+	+	+	+	+	+	+	+	+	-
**Oes5R**	4	+	+	+	-	+	+	+	+	+	-	+	-
**Oes6B(i)**	4	+	+	-	+	+	+	+	+	-	+	+	-
**Oes6B(ii)**	4	+	+	+	+	+	+	+	+	+	+	+	-
**Oes6R**	5	+	+	+	-	+	+	+	+	+	-	+	-
**Oes7B(i)**	5	+	+	+	+	+	+	+	+	+	+	+	-
**Oes7B(ii)**	5	+	+	+	+	+	+	+	+	+	+	+	-
**Oes7R**	5	+	+	+	+	+	+	+	+	+	+	+	-
**Oes8B**	5	+	+	-	-	+	+	+	+	-	-	+	-
**Oes8R**	5	+	+	+	+	+	+	+	+	+	+	+	-
**Oes9B**	4	+	+	-	+	+	+	+	+	+	-	+	-

Footnote: Abbreviations: B: chemo-naïve biopsy sample, R: chemo-exposed resection sample, (i): first biopsy (ii): second biopsy, cyto: cytokeratin.

### The 3D-TGA using close-to-patient cells with mesenchymal cell co-culture accurately models clinical chemo-sensitivity

**Figure 3:**
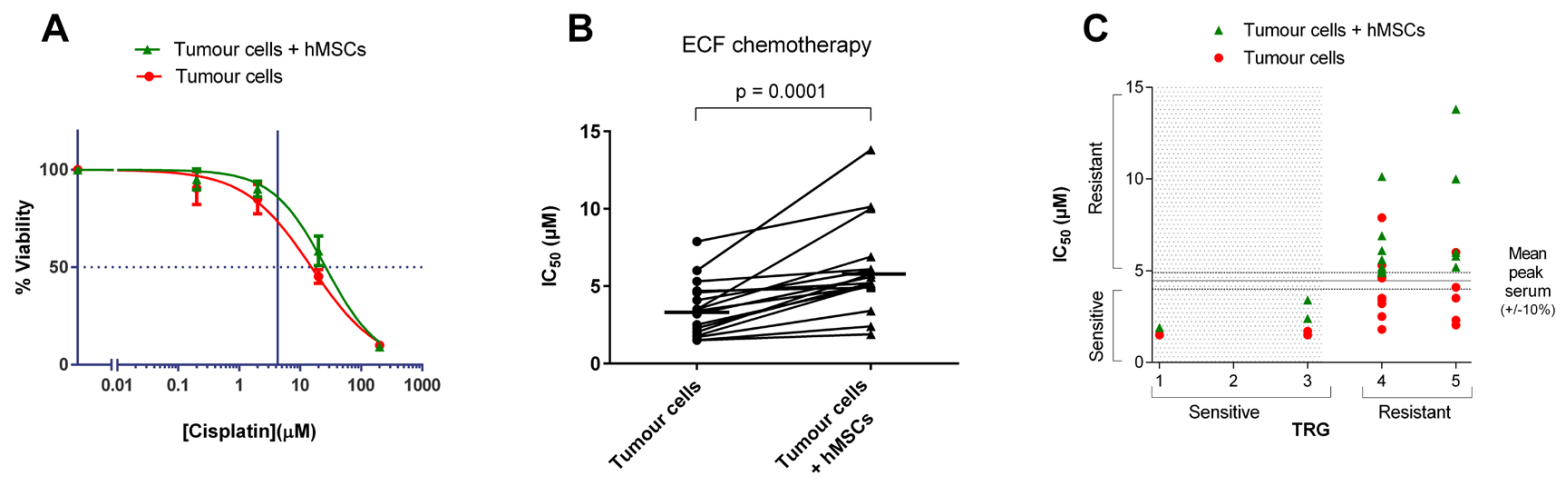
Chemotherapeutic sensitivity profiles Chemo-sensitivity of close-to-patient cancer cell clusters was determined in 3D-TGA, after 4 day exposure to drug combinations at a range of concentrations, using the alamarBlue assay to measure viability. Viability curves were generated and IC_50_ values calculated using GraphPad Prism. **A**. Viability curve for Cisplatin monotherapy. Error bars represent one standard deviation. The horizontal broken line indicates the IC_50_, and the vertical line represents the mean Cisplatin peak serum concentration in patients (4.3 μM). **B**. Sensitivity to ECF in the presence and absence of hMSCs. Horizontal lines represent mean IC_50_s for each group. **C**. Predicted chemo-sensitivity based on 3D-TGA IC_50_ measurement of ECF treatment is compared with the clinical response as reported by the TRG. The 3D-TGA IC_50_ data points, above the mean peak serum delivered to patients, are designated as resistant to chemotherapy. Data points that lie within 10% of the mean peak serum are defined as borderline.

**Figure 4:**
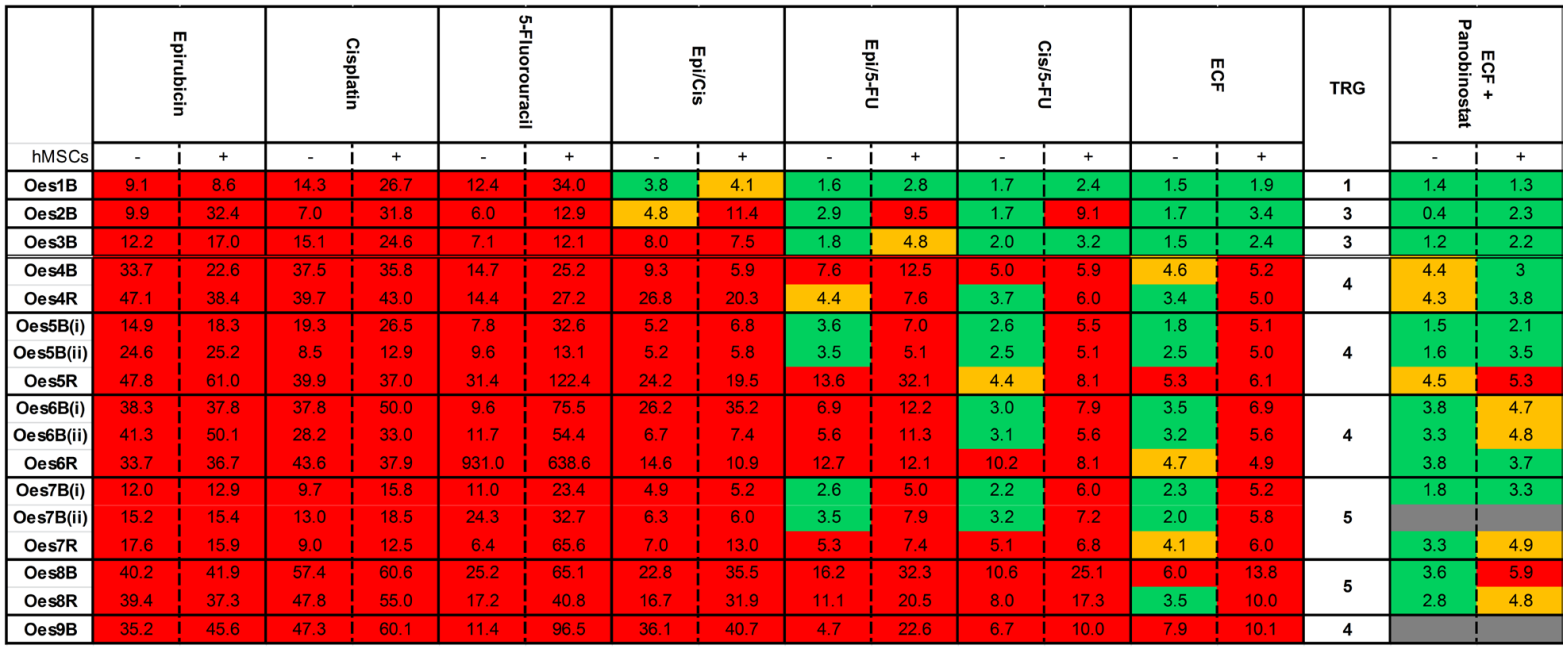
Panel of Response to Chemotherapy Agents Each individual patient's cancer cells were grown in the 3D-TGA with (+) or without (-) the presence of hMSCs, and then underwent pharmacological assessment, to obtain individual patient 3D-TGA IC_50_s, for mono and combination chemotherapy. The patient cancer cell clusters were classified as sensitive (green), borderline (orange), or resistant (red) by comparison of IC_50_ values to mean peak serum concentrations achieved in patients at the doses used in UK clinical practice.

When the 3D-TGA was used to assess chemo-sensitivity, dose-dependent responses were observed (Figure 3A) with IC_50_s that varied between different patients, and sensitivity to chemotherapy agents was reduced when mesenchymal cells were incorporated into the assay (Figure 3B), with these effects being more pronounced when doublet and then triplet chemotherapy was administered (see online Supplementary Figure S3). To investigate whether this finding reflected the clinical chemotherapy response, correlation between the IC_50_s of the individual patient cells grown in the 3D-TGA were determined, in the presence or absence of mesenchymal cells, with the patient's TRG (Figure 3C). When mesenchymal support was present, 100% (*n* = 14) of the TRG 4-5 cancers had IC_50_s higher than the mean peak serum threshold, and 100% (*n* = 3) of the TRG 1-3 cancers had IC_50_s lower than the mean peak serum threshold (Figure 4). Without mesenchymal cells incorporated in the 3D-TGA model, the assay had no predictive value: although all of the TRG 1-3 cancer IC_50_s remained lower than the mean peak serum, only 18% (*n* = 3) of the TRG 4-5 cancers had IC_50_s higher than the mean peak serum threshold.

### Drug efficacy increases in combination

**Figure 5:**
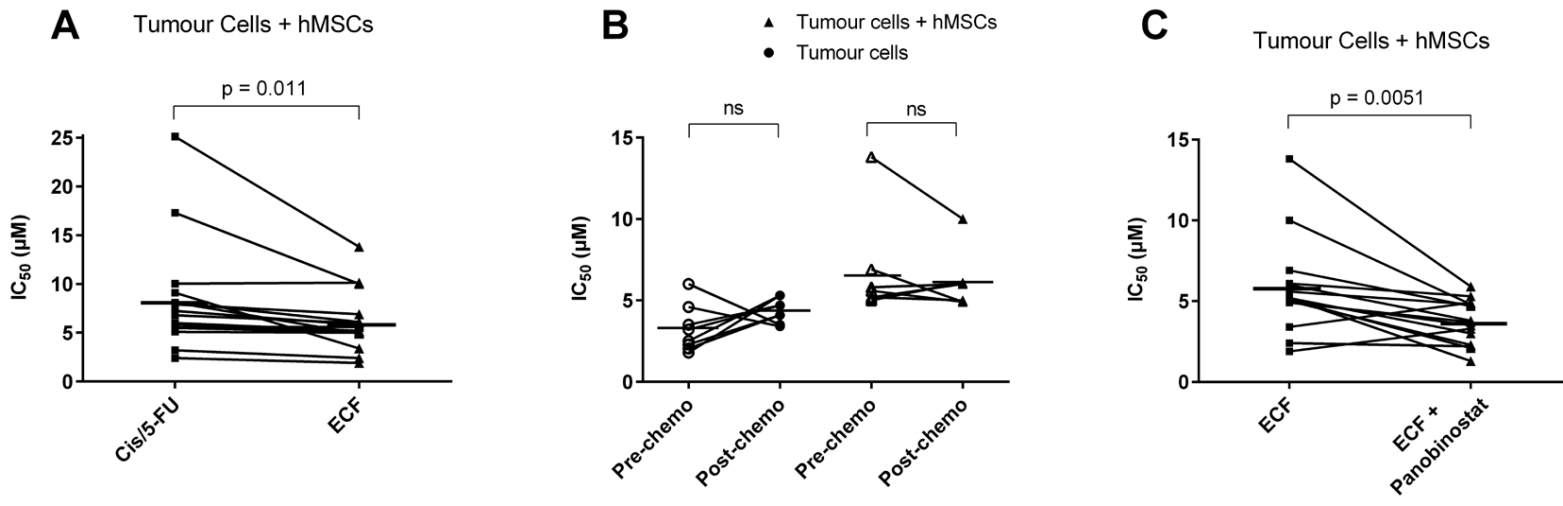
Chemotherapeutic Drug Combinations Chemo-sensitivity of close-to-patient cancer cell clusters was determined in 3D-TGA, to drug combinations at a range of concentrations, using the alamarBlue assay to measure viability. IC_50_ values from viability curves were calculated using GraphPad Prism. **A**. Sensitivity to Cisplatin/5-FU doublet compared to ECF triplet in the presence of hMSCs. **B**. Sensitivity to ECF chemotherapy pre- and post-neoadjuvant chemotherapy, in 3D-TGA models with or without hMSCs. **C**. Sensitivity to ECF chemotherapy compared to ECF in combination with Panobinostat, in the presence of hMSCs. Horizontal lines represent mean IC_50_s.

By examining each drug individually, and their combinations in the 3D-TGA, it is possible to put together a more comprehensive picture of their efficacy, including their individual contribution to the overall effect of the ECF regimen (Figure 4). Individual drugs at the concentrations delivered to patients were never effective; the best drug doublet was Cisplatin/5-Fluorouracil, and further addition of Epirubicin provided an overall marginal gain, with a mean IC_50_ 1.4-fold lower for the ECF triplet than the CF doublet (*p* < 0.05) (Figure 5A).

### Chemo-sensitivity was unchanged pre and post neo-adjuvant chemotherapy

Matched patient samples obtained pre- (chemo-naïve) and post- (chemo-exposed) neoadjuvant chemotherapy were all from patients (*n* = 5) with TRG 4-5 cancers, with remnant tumour bulk at surgical resection. Analysis showed no significant difference in IC_50_ chemo-sensitivity in the 3D-TGA, before or after neo-adjuvant chemotherapy, whether they were grown with or without mesenchymal support (Figure 5B).

### The novel HDACi Panobinostat provides enhanced chemo-sensitivity

Panobinostat was efficacious as a single agent, and while growth with mesenchymal support increased resistance by 1.3-fold (*p* < 0.05), IC_50_s remained within achievable serum concentrations and were thus still classified as sensitive (see online Supplementary Figure S4). When Panobinostat was combined with the SOC triplet ECF chemotherapy, it resulted in a significantly enhanced efficacy (*p* < 0.05) (Figure 5C). Of the 5 patients (12 samples) that were ECF-resistant, only 2 samples remained resistant when subjected to ECF + Panobinostat in combination (Figure 4).

## DISCUSSION

We have developed a novel method of establishing cancer cells from small biopsies of individual patient oesophageal cancers, and then assessing growth in a tumour micro-environment (TME) -relevant 3D assay which enables direct chemo-sensitivity testing and screening of novel drugs. Mesenchymal co-culture had a significant effect; enhancing growth and chemotherapy drug resistance in the 3D model, resulting in accurate prediction of chemo-sensitivity for individual patients.

We present here the first published evidence of a reliable technique to establish individual close-to-patient OAC cells and grow them in a TME-relevant assay, without having to artificially induce immortality (e.g. using a telomerase approach [20, 21]). Although requiring careful attention to detail, the method does not require specialised equipment or materials found outside of a normal tissue culture laboratory, and could be scaled to high-throughput processing, [7] to screen for novel chemotherapeutics effective against this commonly resistant carcinoma. This is a significant step forward towards the goal of delivering personalised health care and personalised oncological therapy in particular. As well as their use for studying chemo-resistance, there are many other promising applications for 3D culture including the study of the cell biology of individual cancers, their similarities, cellular interactions and the relationship to the phenotypical heterogeneity of the disease as a whole.

The characteristics of the established lines matched those of the original patient tissues well, and interestingly, in the case of TFF3 (a functional protein secreted from the apical membrane of established mucosa [22]) demonstrated the requirement for a representative 3D setting. The selective expression of TFF3 in a 3D TME with mesenchymal support (either *in vivo* or *ex vivo*) would suggest that a resumption of apical-basal polarisation, cell-cell interactions and environmental cues (which are absent in monolayer culture) are required for these close-to-patient cells to return to functional status. This reinforces the importance of representative 3D culture, as has been shown in other tissue types, [23] for the production of relevant assay results.

The increased chemo-resistance in the presence of mesenchymal cells in the 3D-TGA underlines the overall importance of the stromal component of the TME, and paracrine interaction between the tumour epithelial cells and their support cells, and the importance of including them in both *in vitro* and *in vivo* assays for pre-clinical drug screening. This study and previous work published by our group [5] shows that the role of the stromal component of the TME in influencing the chemo-resistance is both drug- and individual tumour-dependent. This variation is a more accurate reflection of the diverse clinical outcomes to chemotherapy treatment, where OAC patients individually display variable magnitudes of response to individual drugs and the SOC therapy. [10]

The 3D assay using close-to-patient cancer cells co-cultured with mesenchymal cells provides a clinically-relevant assessment of patient sensitivity to the ECF chemotherapeutic agents, accurately predicting individual clinical chemo-therapeutic response with a sensitivity and specificity of 100%. Although numbers are small, this is a particularly exciting finding, as attempts to correlate laboratory chemo-therapeutic outcomes to individual patient clinical response and provide personalised chemotherapy have previously been unsuccessful. [24, 25] Corresponding survival data would be helpful for clinical application, but has not been reported as it is immature and remains difficult to interpret for a group of this size.

Using standard chemotherapy agents alone, the drug triplet of ECF was the most effective regimen evaluated in the 3D-TGA, closely followed by the Cisplatin/Fluorouracil (5-FU) doublet, which reflects the apparent increased efficacy of the peri-operative ECF triplet over the pre-operative Cisplatin/5-FU doublet observed in clinical practice. [26] However in a head-to-head clinical evaluation, the marginal gain in chemotherapy-efficacy seen from addition of Epirubicin to Cisplatin/5-FU (improved TRG, progression & disease free survival, but not overall survival) is offset by both a significant increase in the toxic side-effects from receiving triplet chemotherapy, and a reduced number of patients completing chemotherapy cycles. [27] This oesophageal 3D-TGA, therefore, has a key role to play: for example, in this study the 3D-TGA results suggest that patients Oes1 and Oes3 may not require the addition of Epirubicin to the Cisplatin/5-FU doublet to achieve sensitivity, whilst patient Oes2 may require triplet ECF chemotherapy to accomplish chemo-sensitivity (Figure 4). The oesophageal 3D-TGA not only allows identification of patients who will potentially not benefit from the SOC chemotherapy, but those that may benefit from a tailored chemotherapy regimen, with reduced exposure to unnecessary or ineffective drugs and their associated side-effects.

The absence of change in chemo-sensitivity between chemo-naïve and matched chemo-exposed patient samples was initially surprising, since the neoadjuvant chemotherapy might be expected to select for increased chemo-resistant clones. However, the pre- and post-treatment samples tested were all from patients in which the primary tumours were graded TRG 4-5 and therefore already chemo-resistant. The 3D-TGA result is thus reflective of the clinical observation that patients who do not respond to neoadjuvant chemotherapy (with a TRG of 4-5) do not have improved survival outcomes with further rounds of the same chemotherapy post-operatively. [10] Although there was no material change in chemo-sensitivity, three of the five patients who had samples obtained pre- and post-chemotherapy (including one patient with 2 biopsy samples taken) had mismatched CD44/ ALDH expression pre- and post-chemotherapy. This could be influenced by clonal selection following exposure to neoadjuvant chemotherapy, and may also be indicative of the time interval and spatial heterogeneity within the tumour tissue itself.

The 3D-TGA can also be used to assess novel drugs, alone or in combination with SOC, across a panel of individual patient cancer cells, for potential translation into clinical practice. Panobinostat is a novel HDACi which has recently undergone accelerated FDA approval for use in myeloma [28]. It is also in multiple phase II and phase III trials currently, as it has a broad spectrum of action against common cancers, [29] at a lower dose than the other current FDA/EMA-approved HDACi. [30] We tested Panobinostat in the oesophageal 3D-TGA, as it has been shown to be effective in a range of solid tumours [29], and in combination with ECF because it has been shown to potentiate the action of anthracyclines (e.g Epirubicin), [31] which are part of the current SOC therapy. The strikingly improved efficacy against oesophageal tumours in our 3D-TGA, shown by combining Panobinostat with the SOC treatment is an exciting prospect. It converted 50% of the previously chemotherapy resistant patient tumour samples into chemo-sensitive responders, suggesting that a future clinical regimen with a HDACi such as Panobinostat, may provide an avenue for more successful global treatment of this frequently chemo-resistant tumour type.

Despite the overwhelming evidence that *in vitro* 3D tumour cell cultures more accurately reflect the complex *in vivo* TME than simple two-dimensional cell monolayers (with respect to gene expression profiles, signalling pathway activity and drug sensitivity [32]), there is still a need for further development and refinement to achieve more accurate 3D cell models of disease, for more clinically-relevant drug screening and in particular using close-to-patient tumour cells and incorporating supporting stromal cells. [33] Using close-to-patient cells is important as it has been shown that the multi-drug resistance transcriptome of cancer cell-lines, bear more resemblance to each other, regardless of the tissue of origin, than to the clinical tumour samples that they are meant to represent. [34] Organotypic modelling, [35, 36] xenografts, [37, 38] and spheroid culture [16, 39] have all been attempted to study oesophageal cancer in a more relevant setting, and in some cases using cancer cells taken directly from the patient. [40, 41] The organoid culture system has also shown much promise for modelling the stem cell niche, particularly in colorectal cancer. [42] However, we have demonstrated the importance of including human stromal mesenchymal cells in the context of studying drug sensitivity, and this is frequently missing in cancer models used for drug discovery. [43]

Although mesenchymal cell induced cancer progression and chemo-resistance has been previously reliably described in both different environments and tumour types, there is a lack of conclusive evidence about the chief mechanisms by which stromal cells (such as mesenchymal and cancer associated fibroblast (CAF) cells) induce these effects [44]. Stroma-cancer cell interactions can be broadly considered as direct cell contact [45–48], secreted signalling factors [49–51], or hypoxia-driven [52, 53]. Clearly although MSCs are potent mediators of resistance to chemotherapy, the key factors and mechanisms in the tumour microenvironment (including in OAC), have yet to be fully identified. This maybe because the cross-talk between stromal cells and cancer cells is complex and context-dependent, may differ between micro-environments and cancer types, and be adaptive in response to the selection pressure applied by chemotherapeutics [54].

We believe that the 3D-TGA model described here-in, using close-to-patient epithelial tissue, a humanised TME, biologically-active 3D matrix and mesenchymal stromal support cells, reproduces some of the key micro-environmental components of the human tumour. Other cell types (for example, cells of the immune system and vascular endothelial cells) may also provide important signals in the case of some individual tumours, so further refinement will be required to model other influences on drug sensitivity or oesophageal tumour biology. However, the correlation between the *in vitro* 3D-TGA assessment of chemo-sensitivity and the observed clinical response described here, demonstrates that in its current format, it is already a useful tool. To demonstrate its clinical applicability for providing tailored treatment for individual patients, our findings will require confirmation in an expanded cohort. However, in its current format, this novel method of expanding individual patient oesophageal cancer cells in the laboratory and using them for drug screening, has potential for both reducing the use of animals in the early stages of drug development (due to availability of a more clinically-relevant *in vitro* assay), and has potential to have a significant impact on clinical outcomes by enabling accurate identification of new treatments, in a more cost-effective manner, including those targeting the stroma.

## MATERIALS AND METHODS

### Ethics statement

Investigation has been conducted in accordance with the ethical standards and according to the Declaration of Helsinki and according to national and international guidelines and has been approved by the authors’ institutional review board.

### Establishing close-to-patient cells using a feeder layer method

Endoscopic tumour biopsies (REC: 10/H0401/80) and fresh surgical specimens (REC: 10/H0405/6) were collected with informed consent from patients at the Nottingham University Hospitals NHS Trust in 2014-2015, and used in accordance with National Research Ethics Service approval. Chemo-naïve tumour biopsies were taken from patients (Oes1B, Oes2B etc) undergoing standard oesophageal cancer endoscopic staging examinations, either at oesophagogastroduodenoscopy or at endoscopic ultra-sound, or both; resulting in two chemo-naïve samples from the same patient (designated for example as Oes5B(i) for first biopsy, Oes5B(ii) for second biopsy). If disease stage allowed treatment with curative intent, patients subsequently underwent routine neo-adjuvant chemotherapy (3 cycles of anthracycline, platinum and fluoropyrimidine) before definitive surgical resection of their oesophageal tumour. A matching chemotherapy-exposed tumour specimen was collected at surgical resection where possible (designated as Oes R). All resected tumours were examined by a dedicated team of Consultant Gastrointestinal Histopathologists, and allocated a TRG from 1-5, as described by Mandard (see online supplementary table S1). This histopathological grading of chemotherapy response in oesophageal cancer, (where TRG 1-3 cancers are considered chemo-sensitive and TRG 4-5 chemo-resistant) directly relates to prognosis. [3, 55]

The tissue specimens were transferred from the hospital to the laboratory, and then processed and disaggregated to produce a cellular suspension (see online supplementary method S1). *In vitro* tumour cell growth was then established and expanded with a layer of supporting feeder cells according to the method of Liu *et al*. [56] From this early passage material, the tumour epithelial cells were expanded and harvested separately from the cancer associated fibroblasts using differential trypsinisation. Tumour cell number and viability was determined using trypan blue exclusion and analysed by flow cytometry for expression of the epithelial marker, EpCam (see online supplementary method S2). At less than passage 5, cell aliquots were cryopreserved, utilised for the 3D-TGA, xenografted to study tumourigenicity (see online supplementary method S3), and formalin fixed before being embedded in agarose for immunohistochemical analysis.

### Study inclusion criteria

Tumour biopsies were collected from patients early in their diagnostic and staging pathway, however due to subsequent staging with advanced disease, poor performance status preventing chemotherapy, or intolerance of the chemotherapy regimen, two-thirds of these patients did not undergo the full curative therapy regimen, and therefore did not have a clinical chemotherapy response to compare with the *ex vivo* result. The following inclusion criteria for pharmacological assessment in the 3D-TGA were required to ensure that the study population was undergoing comparable curative treatment: a) adenocarcinomas of the lower oesophagus or gastro-oesophageal junction (GOJ); b) completed all 3 cycles of neo-adjuvant chemotherapy, without dose reductions; c) underwent definitive surgery and were assigned a TRG; d) an *in vitro* patient line was established for the paired chemo-exposed tumour (where tissue was available).

### 3D-tumour growth assay

The individual patient's epithelial cells, were co-cultured in the 3D-TGA with or without human mesenchymal stem cells (hMSC) in a modified Cultrex^®^ basement membrane extract (BME) (Trevigen, MD, USA), using serum-free conditions, with a human tissue reflective pH and glucose (see online supplementary method S4), as previously reported. [5] Cellular metabolism and growth was assessed with an alamarBlue^®^ cell fluorescence assay (ThermoFisher Scientific, Loughborough, UK) and measured on a fluorescent plate reader (excitation 560 nm, emission 588 nm, Flex Station II, Molecular Devices).To enable separate assessment of the co-cultured cells within the 3D-TGA, hMSCs constitutively expressing the red fluorescent protein mCherry were generated (see online supplementary method S5). The mCherry-labelled hMSC component of the 3D-TGA was measured separately by the fluorescent plate reader (excitation 553 nm, emission 613 nm).

### Pharmacological assessment

The standard chemotherapy regimen used for oesophageal adenocarcinoma (OAC) in the UK and administered to the patients in this study, is Epirubicin, Cisplatin and 5-Fluorouracil/Capecitabine (ECF) pre- and post-operatively, [2] and so this regimen was replicated in the 3D-TGA (see online supplementary table S2). The novel histone deacetylase inhibitor (HDACi) drug, Panobinostat, was also assessed to evaluate the 3D-TGA as a potential platform for appraisal of new drugs, with assessment of new drug efficacy and in combination with the current ECF standard-of-care (SOC), for potential translation into clinical practice. Following the addition of drugs to the cancer cell clusters in the 3D-TGA, the chemo-toxic effect was calculated as a percentage of the matched untreated control. IC_50_ curves were generated for the drugs both individually and in combination, as previously described by our group, [5] using the Chou-Talalay method. [57] The mean peak serum concentration achieved in patients for each drug (Epirubicin 4.5 μM, Cisplatin 4.3 μM, 5-Fluorouracil & Capecitabine 4.6 μM; see online supplementary table S2) was compared with the IC_50_ values, and chemo-response was thus defined as sensitive, borderline (+/− 10% of the mean peak serum), or resistant.

### Imaging and staining of the 3D-TGA

Serial bright-field and immunofluorescent images of the co-cultured mCherry-labelled hMSCs and cancer cell clusters were obtained at day 0, 3 and 7, using a Nikon Eclipse Ti-E microscope with differential interference contrast (DIC). Forty z-stacks were taken throughout the depth of the well, and extended depth of field projection picture was produced using the Nikon NIS Elements software, allowing a condensed view of the three-dimensional profile of the cancer cell clusters. On day 7, cell clusters from the 3D-TGA were extracted using the standard method, [58] and immuno-stained as previously reported, [5] with Trefoil Factor 3 (TFF3, Abcam), AlexFluor488 (ThermoFisher Scientific) and mounted in Prolog Gold Anti-fade containing DAPI (ThermoFisher Scientific). Immunofluorescent images of the stained clusters were obtained with the Nikon Eclipse Ti-E microscope and NIS Elements Advanced Research Software.

### Immunohistochemistry characterisation

Tumour tissue and the agarose-embedded close-to-patient cancer cells were formalin fixed and paraffin embedded (FFPE) before 4 μm sections were cut for both H&E and immunohistochemistry (IHC) analysis. IHC was performed using standard techniques and in line with the manufacturer's instructions for the following primary antibodies: Cytokeratin (MNF116, DAKO), EpCam (Ber-EP4, DAKO), CD44 (DF1485, DAKO), p53 (DO-7, DAKO), Vimentin (V9, DAKO), TFF3 (Abcam), and ALDH1A1 (EP1933Y, Abcam) (see online supplementary method S6). Sections were viewed with a Leica DMLB Bright-field Microscope (Leica-microsystems, Milton Keynes, UK) and images acquired with Leica QWin Standard v3 software. The presence of any characteristically stained cells was considered positive with respect to negative controls, and confirmed by a second blinded individual.

### Statistical analysis

Two-way ANOVA was performed to compare the different parameters among the different groups when assessing the relative efficacy of the drug combinations. The *t*-test was used to calculate the significance of difference between paired groups, and Mann-Whitney U test between independent groups, with a significance level of *p* < 0.05. Statistics were computed with GraphPad Prism 5 Software (San Diego, CA, USA) and plotted with mean values, and error bars for standard deviation.

## SUPPLEMENTARY MATERIALS FIGURES AND TABLES





## Abbreviations

3D-TGA: Three Dimensional Tumour Growth Assay
TME: Tumour Micro Environment
TRG: Tumour Regression Grade
GOJ: Gastro Oesophageal Junction
hMSC: Human Mesenchymal Stem Cells
BME: Basement Membrane Extract
OAC: Oesophageal Adenocarcinoma
ECF: Epirubicin / Cisplatin / Fluorouracil
HDACi: Histone Deacetylase inhibitor
SOC: Standard of Care
TFF3: Trefoil Factor 3
FFPE: Formalin Fixed Paraffin Embedded
IHC: Immunohistochemistry
CSC: Cancer Stem Cell
