# Supplementary material for: Individual patient oesophageal cancer 3D models for tailored treatment
Source: Oncotarget. 2016 Oct 6;8(15):24224–36. doi: 10.18632/oncotarget.12500 (PMC5421842; doi:10.18632/oncotarget.12500)
Supplement: Supplementary file 1 [file oncotarget-08-24224-s001.pdf]

Individual patient oesophageal cancer 3D models for tailored treatment

Supplementary Material

ONLINE SUPPLEMENTARY FIGURES

(see following pages)

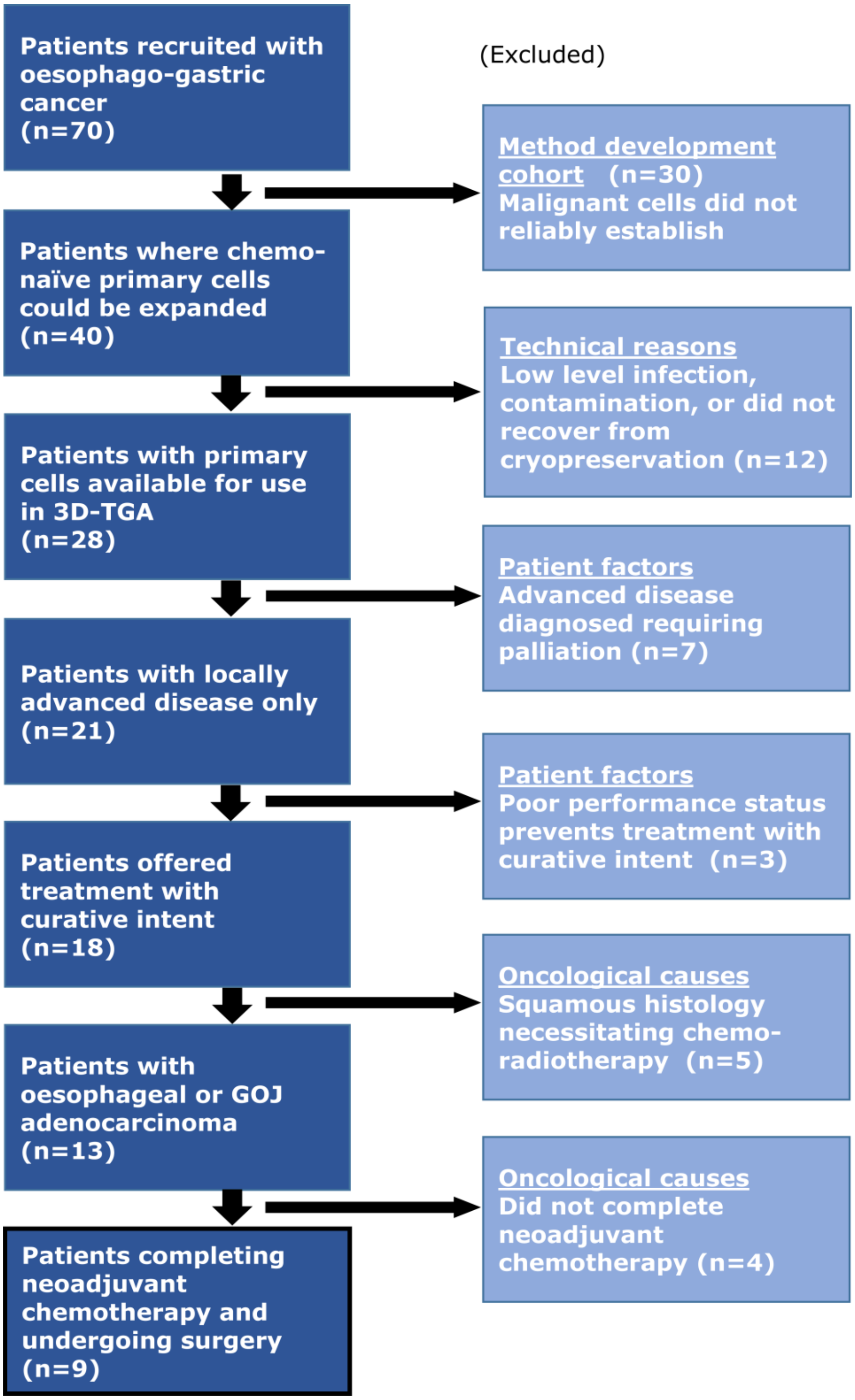

Figure S1: Application of Inclusion Criteria to recruited Study Patients.

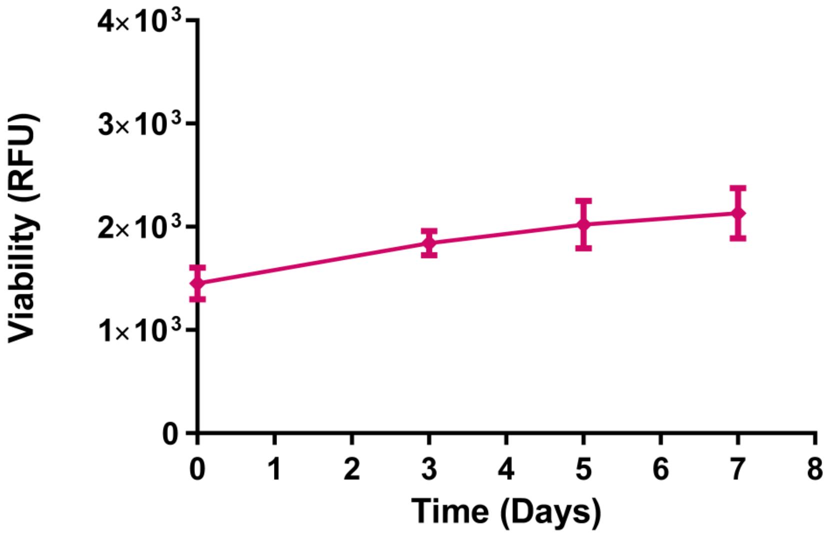

Figure S2: Growth of the mCherry-labelled hMSC Component.

Growth of the mCherry-labelled hMSC stromal component of the 3D-TGA was measured independently from the epithelial cancer cluster growth, using the mCherry fluorescent signal (RFU).

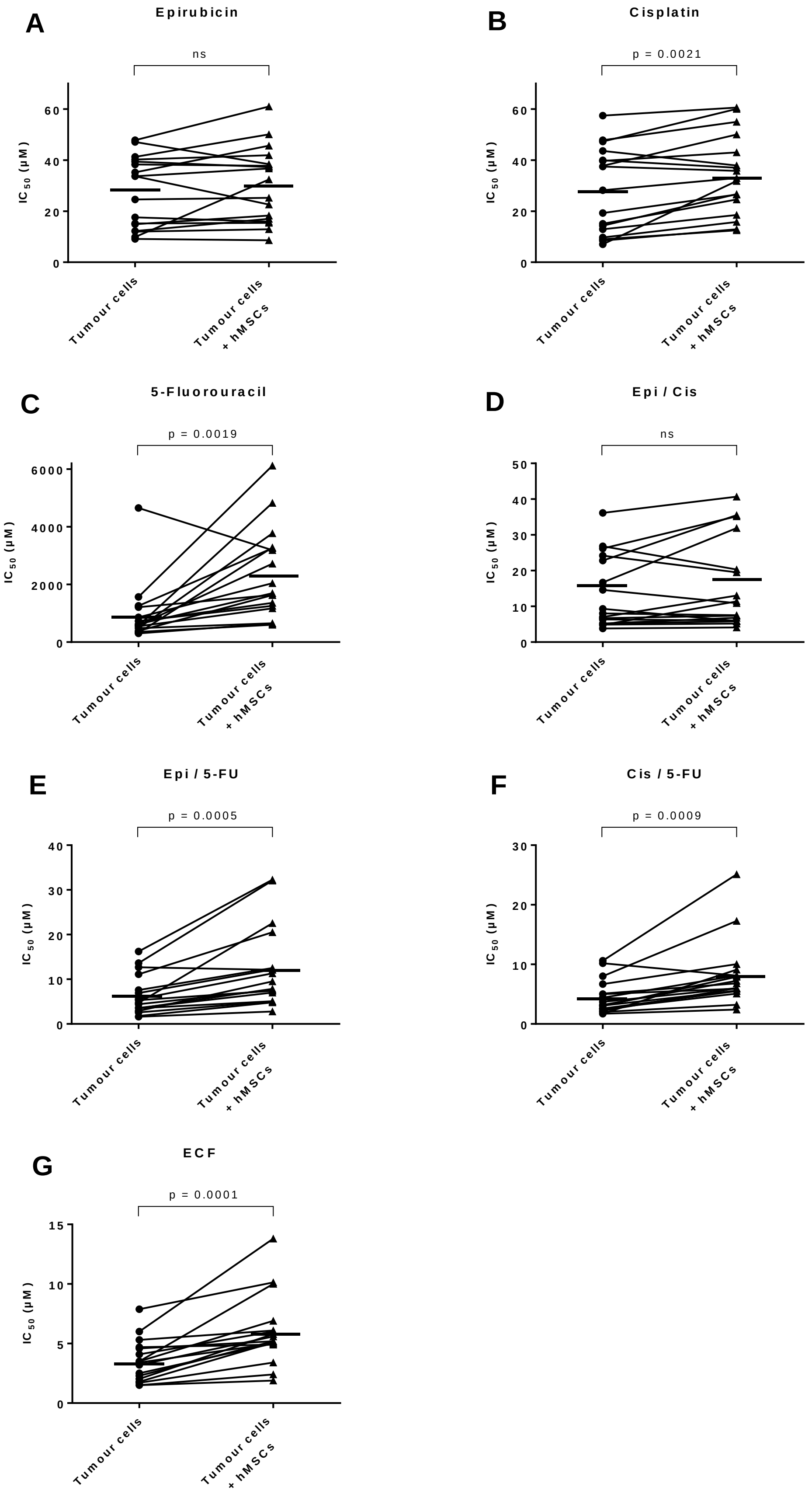
A
Epirubicin
ns
IC50 (µM)
0
20
40
60
Tumour cells
Tumour cells + hMSCs
B
Cisplatin
p = 0.0021
IC50 (µM)
0
20
40
60
Tumour cells
Tumour cells + hMSCs
C
5-Fluorouracil
p = 0.0019
IC50 (µM)
0
2000
4000
6000
Tumour cells
Tumour cells + hMSCs
D
Epi / Cis
ns
IC50 (µM)
0
10
20
30
40
50
Tumour cells
Tumour cells + hMSCs
E
Epi / 5-FU
p = 0.0005
IC50 (µM)
0
10
20
30
40
Tumour cells
Tumour cells + hMSCs
F
Cis / 5-FU
p = 0.0009
IC50 (µM)
0
10
20
30
Tumour cells
Tumour cells + hMSCs
G
ECF
p = 0.0001
IC50 (µM)
0
5
10
15
Tumour cells
Tumour cells + hMSCs

Figure S3: Influence of Mesenchymal Cells upon Chemo-resistance

The 3D-TGA model demonstrates significantly higher chemo-resistance in the models incorporating mesenchymal support in a drug and patient dependent manner.

Sensitivity of close-to-patient cells was determined in 3D-TGA, after 4 day exposure to drug combinations at a range of concentrations, using the alamarBlue assay to measure viability. Viability curves were generated and IC_{50} values plotted using GraphPad Prism. Sensitivity to the SOC chemotherapy drugs alone (**A, B, C**), and their doublet combinations (**D, E, F**) is demonstrated with and without co-culture with mesenchymal support. The significance of the magnitude of mesenchymal induced chemo-resistance increases and is maximal with the triplet combination chemotherapy (**G**).

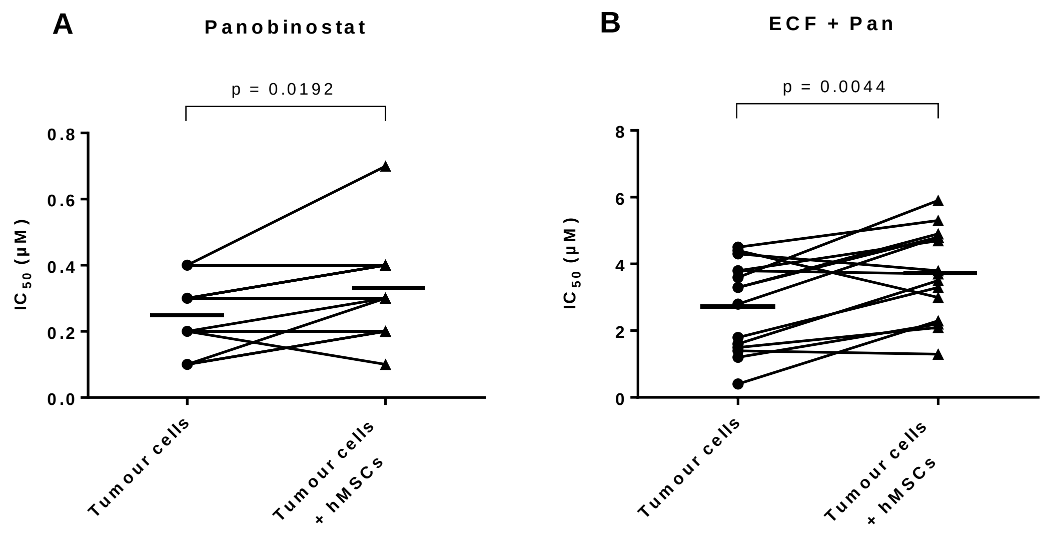

Figure S4: Combination Chemotherapy with Panobinostat provides Enhanced Chemo-sensitivity

There is significantly greater chemo-resistance to Panobinostat alone, and in combination with ECF chemotherapy, when 3D-TGA models incorporate mesenchymal support.

Sensitivity of close-to-patient cells was determined in 3D-TGA, with and without mesenchymal cell co-culture, after 4 day exposure to drug combinations at a range of concentrations, using the alamarBlue assay to measure viability. Viability curves were generated and IC_{50} values determined using GraphPad Prism. Sensitivity to Panobinostat alone and in combination with standard of care ECF chemotherapy were plotted for assays with and without mesenchymal cell co-culture. Increased resistance to Panobinostat monotherapy (**A**) and in combination with ECF (**B**) is demonstrated in the presence of mesenchymal support.

ONLINE SUPPLEMENTARY METHODS

S1: Tissue Transfer and Disaggregation

Fresh endoscopic biopsy samples and surgical material was immediately placed into tissue transfer media (Dulbecco's modified Eagle's medium with 10% foetal bovine serum, 1% 0.2 M L-glutamine, 1% penicillin / streptomycin / amphotericin B, 0.1% 1 mg/ml hydrocortisone, 0.75% 1 mg/ml insulin) at 4°C, and transferred to the laboratory, and processed within 4-6 hours.
On arrival at the laboratory, the tumour material was dissected; the majority of the tissue was finely minced for live use, and further small portions were either formalin-fixed and paraffin-embedded (FFPE) for immunohistochemistry (IHC), or stored in RNA*later* (Ambion) for subsequent analysis. The finely-minced tumour was disaggregated using type II collagenase (100 U/ml, Gibco, ThermoFisher Scientific) and dispase (2.4 U/ml, Gibco, ThermoFisher Scientific) in Hank’s balanced salt solution (HBSS) without calcium or magnesium (Sigma) at 37°C under constant rotation. Cells were removed at 1-2 hourly intervals until the tumour was completely disaggregated.

S2: Flow Cytometry

The expanded individual patient tumour cells were stained with anti-human EpCam-Alexa Fluor 647 mouse monoclonal antibody (VU1D9, Signalling Technology, USA) and corresponding isotype control (Clone MOPC-21). Cells were washed and fixed in 2% formaldehyde and analysed on a Becton Dickinson LSRII flow cytometer, equipped with a 488 nm laser. Data was analysed with Weasel software Version 3.0.2 (The Walter and Eliza Hall Institute of Medical Research, Melbourne, Australia).

S3: Murine xenograft Tumourigenicity Assay

Individual patient cancer epithelial cells ($2x10^6$ cells) were admixed (in a 2:1 tumour:stroma ratio) with low passage bone marrow-derived mesenchymal stem cells (hMSCs) ($1x10^6$ cells) (ScienCell) in 100 µl medium and suspended in 100 µl cold Matrigel (BD Biosciences, Germany). This was injected subcutaneously into the right and left flanks of sex-matched 6-8 weeks old MF-1 nude or RAG mice (Harlan).

Animal procedures were carried out with ethical approval under UK Home Office Licence (PPL 40/3559) in accordance with the 3R’s framework for humane animal research. Tumour growth from the cell injections was monitored by caliper measurements, until they had reached the maximum acceptable size, when tumours were surgically removed under anaesthesia and portioned for histological examination, cryopreservation and re-implantation.

S4: 3D-TGA Method

The 3D-TGA method was performed as previously described,[1, 2] and adjusted to take account of the 384-well plate format (for 6 repeated wells per condition) and the higher concentration of modified Cultrex BME. In brief; $6.25x10^3$ tumour cells and $3.125\ x10^3$ mesenchymal cells (2:1 tumour: stroma ratio) were admixed and seeded in 12.5 µl cold BME per well at 12 mg/ml, in a low-adherent, black-walled, clear-bottom, 384-well plate

(Corning, New York, USA). Once embedded in the 3D matrix and brought to room temperature, a media overlay of 12.5 µl per well was used for cell clusters nutrition. Plates were incubated for 72 hours to allow cells to establish within the extracellular matrix and form cell clusters. These were then treated with increasing concentrations of the chemotherapy drugs as a 12.5 µl media overlay per well, to enable endpoint drug sensitivity. Following 96 hours exposure, growth was assessed using a 12.5 µl per well overlay alamarBlue® assay, and evaluated using a fluorescent plate reader.

S5: Establishment of mCherry-labelled hMSCs

Low passage hMSCs were seeded in 6-well plates ($2x10^5$ cells/ well) and left to adhere overnight before being transduced with mCherry (Clontech Lentiviral X containing mCherry under the control of CMV_{IE} promoter), according to the manufactures instructions. Transduced cells were selected by exposing cells to puromycin at a concentration shown to kill parental cells (1µg/ml). Selection was maintained for a week. Surviving cells were expanded, expression of mCherry determined by flow cytometry, and aliquots of the low passage mCherry-labelled hMSCs cryopreserved for subsequent use.

S6: IHC Methodology

Following optimisation, staining was undertaken in batches for each antigen with the corresponding negative controls, in line with previous reports in the published literature. In brief; slides were de-paraffinised and underwent heat induced epitope retrieval. Following equilibration in phosphate-buffered saline (PBS) the endogenous peroxidase activity was blocked by incubating slides in a solution of hydrogen peroxide in PBS. Endogenous proteins were blocked with the serum corresponding to the secondary antibody, before incubation with the primary antibody in question. Amplification and visualisation was then performed either with streptavidin-biotin-peroxidase or horseradish-peroxidase (HRP) technique with diaminobenzidine (DAB), which becomes visible as a brown precipitate. Finally, slides were counter-stained with haematoxylin before mounting.

REFERENCES

1. Sasser AK, Mundy BL, Smith KM, Studebaker AW, Axel AE, Haidet AM, Fernandez SA and Hall BM. Human bone marrow stromal cells enhance breast cancer cell growth rates in a cell line-dependent manner when evaluated in 3D tumor environments. Cancer letters. 2007; 254(2):255-264.
2. Onion D, Argent RH, Reece-Smith AM, Craze ML, Pineda RG, Clarke PA, Ratan HL, Parsons SL, Lobo DN, Duffy JP, Atherton JC, McKenzie AJ, Kumari R, King P, Hall BM and Grabowska AM. 3-Dimensional Patient-Derived Lung Cancer Assays Reveal Resistance to Standards-of-Care Promoted by Stromal cells but Sensitivity to Histone Deacetylase Inhibitors. Molecular cancer therapeutics. 2016.
