# Supplementary material for: Individual patient oesophageal cancer 3D models for tailored treatment
Source: Oncotarget. 2016 Oct 6;8(15):24224–36. doi: 10.18632/oncotarget.12500 (PMC5421842; doi:10.18632/oncotarget.12500)
Supplement: Supplementary file 2 [file oncotarget-08-24224-s002.docx]

## ONLINE SUPPLEMENTARY TABLES

### Table S1: Tumour Regression Grades

| Table S1: Mandard Tumour Regression Grading | |
| --- | --- |
| Grade | **Definition** |
| 1 | No residual cancer |
| 2 | Rare residual cancer cells |
| 3 | Increase in cancer cell numbers, but fibrosis predominates |
| 4 | Residual cancer outgrowing fibrosis |
| 5 | Absence of regressive changes |

Defined by Mandard [[1](#_ENREF_1)]

### Table S2: Peak Serum Concentration of Chemotherapy Agents in Humans

| **Table S2: Peak serum concentration of chemotherapy agents in humans** | | | |
| --- | --- | --- | --- |
| **Drug** | **[Mean Peak Serum]** | **Supplementary Reference** | **Dose in Reference** |
| Epirubicin | 4.5 µM | [[2-6](#_ENREF_2)] | 50 mg/m^2^ |
| Cisplatin | 4.3 µM | [[7-9](#_ENREF_7)] | 60 mg/m^2^ |
| 5-Fluorouracil & Capecitabine | 4.6 µM | [[10-12](#_ENREF_10)] | 200 mg/m^2^  & 625 mg/m^2^ |
| Panobinostat | 1.5 μM | [[13](#_ENREF_13)] | 20 mg/m^2^ |

The standard-of-care chemotherapy regimen comprised of 3 cycles of anthracycline, platinum and fluoropyrimidine. Following the 2006 MAGIC trial,[[14](#_ENREF_14)] patients were administered an ECF regimen - Epirubicin, Cisplatin and infusional 5-Fluorouracil. A complementary follow-on trial [[15](#_ENREF_15)] showed that infusional 5-Fluorouricil can be safely substituted for an oral Fluorouracil pro-drug: Capecitabine.[[16](#_ENREF_16)] Given that both regimens have the same gross clinical efficacy,[[15](#_ENREF_15)] for purposes of clarity in this paper they will be all be referred to collectively as ECF chemotherapy.

### Table S3: Oncological and Demographic Characteristics of Recruited Patients

| **Table S3: Oncological and Demographic Characteristics of Patients** | | | |
| --- | --- | --- | --- |
| **Characteristics** | | **Primary cells established** | **Primary cells**  **not established** |
| Patients | | n=28 | n=12 |
| Gender  (number & ratio) | | F= 7, M = 21  0.33 | F = 3, M = 9  0.33 |
| Age (mean) | | 66 | 69 |
| Charleson comorbidity  score (mean) | | 1.4 | 1.6 |
| Performance status (mean) | | 0.5 | 0.6 |
| Known Barretts  (number & percentage) | | 6 (21) | 5 (33) |
| Histology  (number) | | Adenocarcinoma: 25  Squamous: 7  Linitus: 1 | Adenocarcinoma: 10  Squamous: 1  Linitus: 1 |
| Site of tumour  (number & percentage) | | Gastric: 1 (3)  GOJ: 17 (61)  Oesophageal: 10 (36) | Gastric: 2 (17)  GOJ: 8 (66)  Oesophageal: 2 (17) |
| Pre-chemo / staging TNM | cT (mean) | 3 | 3 |
|  | cN (mean) | 1 | 1 |
| Tumour Regression Grade where available (mean) | | 4 | 4 |

## REFERENCES

1. Mandard AM, Dalibard F, Mandard JC, Marnay J, Henry-Amar M, Petiot JF, Roussel A, Jacob JH, Segol P, Samama G and et al. Pathologic assessment of tumor regression after preoperative chemoradiotherapy of esophageal carcinoma. Clinicopathologic correlations. Cancer. 1994; 73(11):2680-2686.

2. Eksborg S, Hardell L, Bengtsson NO, Sjodin M and Elfsson B. Epirubicin as a single agent therapy for the treatment of breast cancer--a pharmacokinetic and clinical study. Medical oncology and tumor pharmacotherapy. 1992; 9(2):75-80.

3. Coukell AJ and Faulds D. Epirubicin. An updated review of its pharmacodynamic and pharmacokinetic properties and therapeutic efficacy in the management of breast cancer. Drugs. 1997; 53(3):453-482.

4. Danesi R, Innocenti F, Fogli S, Gennari A, Baldini E, Di Paolo A, Salvadori B, Bocci G, Conte PF and Del Tacca M. Pharmacokinetics and pharmacodynamics of combination chemotherapy with paclitaxel and epirubicin in breast cancer patients. British journal of clinical pharmacology. 2002; 53(5):508-518.

5. Mayne Pharma Ltd. Epirubicin Hydrochloride for Injection. UK Medicines and Healthcare products Regulatory Agency <http://wwwmhragovuk/home/groups/par/documents/websiteresources/con2025068pdf>. 2006.

6. Lunardi G, Venturini M, Vannozzi MO, Tolino G, Del ML, Bighin C, Schettini G and Esposito M. Influence of alternate sequences of epirubicin and docetaxel on the pharmacokinetic behaviour of both drugs in advanced breast cancer. Annals of oncology : official journal of the European Society for Medical Oncology / ESMO. 2002; 13(2):280-285.

7. Tegeder I, Brautigam L, Seegel M, Al-Dam A, Turowski B, Geisslinger G and Kovacs AF. Cisplatin tumor concentrations after intra-arterial cisplatin infusion or embolization in patients with oral cancer. Clinical pharmacology and therapeutics. 2003; 73(5):417-426.

8. Rademaker-Lakhai JM, Crul M, Zuur L, Baas P, Beijnen JH, Simis YJ, van Zandwijk N and Schellens JH. Relationship between cisplatin administration and the development of ototoxicity. Journal of clinical oncology : official journal of the American Society of Clinical Oncology. 2006; 24(6):918-924.

9. Minami H, Ohe Y, Niho S, Goto K, Ohmatsu H, Kubota K, Kakinuma R, Nishiwaki Y, Nokihara H, Sekine I, Saijo N, Hanada K and Ogata H. Comparison of pharmacokinetics and pharmacodynamics of docetaxel and Cisplatin in elderly and non-elderly patients: why is toxicity increased in elderly patients? Journal of clinical oncology : official journal of the American Society of Clinical Oncology. 2004; 22(14):2901-2908.

10. Saif MW, Choma A, Salamone SJ and Chu E. Pharmacokinetically guided dose adjustment of 5-fluorouracil: a rational approach to improving therapeutic outcomes. Journal of the National Cancer Institute. 2009; 101(22):1543-1552.

11. European Medicines Agency. Xeloda Scientific Product Information. <http://www.ema.europa.eu/docs/en_GB/document_library/EPAR_-_Scientific_Discussion/human/000316/WC500058145.pdf>. 2005.

12. Reigner B, Blesch K and Weidekamm E. Clinical pharmacokinetics of capecitabine. Clinical pharmacokinetics. 2001; 40(2):85-104.

13. Morita S, Oizumi S, Minami H, Kitagawa K, Komatsu Y, Fujiwara Y, Inada M, Yuki S, Kiyota N, Mitsuma A, Sawaki M, Tanii H, Kimura J and Ando Y. Phase I dose-escalating study of panobinostat (LBH589) administered intravenously to Japanese patients with advanced solid tumors. Investigational new drugs. 2012; 30(5):1950-1957.

14. Cunningham D, Allum WH, Stenning SP, Thompson JN, Van de Velde CJ, Nicolson M, Scarffe JH, Lofts FJ, Falk SJ, Iveson TJ, Smith DB, Langley RE, Verma M, Weeden S, Chua YJ and Participants MT. Perioperative chemotherapy versus surgery alone for resectable gastroesophageal cancer. The New England journal of medicine. 2006; 355(1):11-20.

15. Cunningham D, Starling N, Rao S, Iveson T, Nicolson M, Coxon F, Middleton G, Daniel F, Oates J, Norman AR and Upper Gastrointestinal Clinical Studies Group of the National Cancer Research Institute of the United K. Capecitabine and oxaliplatin for advanced esophagogastric cancer. The New England journal of medicine. 2008; 358(1):36-46.

16. Koukourakis GV, Kouloulias V, Koukourakis MJ, Zacharias GA, Zabatis H and Kouvaris J. Efficacy of the oral fluorouracil pro-drug capecitabine in cancer treatment: a review. Molecules. 2008; 13(8):1897-1922.
